# Opium Addiction and Risk of Laryngeal and Esophageal Carcinoma

**Published:** 2017-01

**Authors:** Mehdi Bakhshaee, Hamid Reza Raziee, Reza Afshari, Amin Amali, Mahmoud Roopoosh, Ali Lotfizadeh

**Affiliations:** 1 *Sinus and Surgical Endoscopic Research Center, Faculty of Medicine, Mashhad University of Medical Sciences, Mashhad, Iran.*; 2 *Cancer Research Center, Faculty of Medicine, Mashhad University of Medical Sciences, Mashhad, Iran.*; 3 *Addiction Research Center, Mashhad University of Medical Sciences, Mashhad, Iran*; 4 *Occupational Sleep Research Center, Otorhinolaryngology Research Center, Imam Khomeini Hospital Complex, Tehran University of Medical Sciences, Tehran, Iran.*; 5 *General Medical Practitioner, Mashhad University of Medical Sciences, Mashhad, Iran*; 6 *Volunteer of the Sinus and Surgical Endoscopic Research Center, Faculty of Medicine, Mashhad University of Medical Sciences, Mashhad, Iran.*

**Keywords:** Esophageal carcinoma, Laryngeal carcinoma, Opium, Risk factors

## Abstract

**Introduction:**

Cigarette smoking and alcohol consumption have a well-known effect on the development of upper aerodigestive tract carcinomas, but such a role for opium is questionable. This study was designed to assess the correlation between opium inhalation and cancer of the larynx and upper esophagus.

**Materials and Methods:**

Fifty eight patients with laryngeal cancer, ninety eight patients with upper esophageal cancer and twenty seven healthy individuals with no evidence of head and neck or esophageal malignancies were selected from Otolaryngology and Radiation Oncology Department of Mashhad University of Medical Sciences. Duration and amount of cigarette smoking and opium consumption were recorded through comprehensive interviews.

**Results:**

The crude odds ratio for laryngeal cancer was 5.58 (95% CI 2.05-15.15, P=0.000) in cigarette smokers relative to non-smokers and 9.09 (95% CI 3.21-25.64, P=0.000) in opium users relative to non-users. The crude odds ratio for esophageal cancer was 0.44 (95% CI 0.18-1.09, P=0.07) in cigarette smokers relative to non-smokers and 1.44 (95% CI 0.57-3.62, P=0.43) in opium users relative to non-users. After adjusting for smoking, the odds ratio for laryngeal cancer in opium users relative to non-users was 6.06 (95% CI 1.10-33.23, P=0.05). Laryngeal cancer was detected at a significantly lower age in opium users (54.54±10.93 vs 62.92±10.10 years, P=0.02) than in smokers. This effect was not observed in esophageal cancer. Although the duration (year 17.50±14.84 vs 21.91±14.03; P=0.34) and amount (pack/day 0.625 vs 0.978; P=0.06) of smoking were higher among those who were opium dependent, these differences were not statistically significant (P=0.34 and P=0.06, respectively).

**Conclusion:**

Opium addiction by snuffing is an independent risk factor for the development laryngeal cancer but not esophageal cancer. Cigarette smoking increases this risk. Opium dependency increases the likelihood of developing laryngeal cancer at a younger age.

## Introduction

Illicit drug use is a global phenomenon, which has drastic effects on public health. Among various drugs of abuse, opiates are among the most commonly used substances and pose a large public health problem around the world. In 2009, there were between 12 to 21 million opiate users worldwide. Among various opiates, opium is a commonly abused substance (1). Estimates from 2008 suggest that there are approximately 4 million opium users worldwide, 80% of whom live in Asia. Afghanistan is responsible for greater than half of all global cultivation of opium. Opium from Afghanistan flows through Iran, Pakistan, and Central Asia, where ease of access in addition to traditional beliefs regarding this substance, have resulted in high rates of consumption (2). 

The use of opium is associated with various adverse health effects including different types of cancer. Previous studies have shown an association between opium and oral, bladder, lung, and gastric cancer (3-5). Some reports also suggest a correlation between opium consumption and cancers of the esophagus and larynx (6,7). However, the evidence for such a correlation is limited and the nature of this correlation is not clearly understood. Cancers of the esophagus and larynx are aggressive and often fatal. Cigarette smoking and alcohol abuse are known risk factors for the development of these cancers.

Laryngeal cancer is the most common malignancy of the head and neck and the second most common respiratory tract cancer after lung cancer. In 2008, there were an estimated 150,000 cases of laryngeal cancer world wide and 80,000 deaths from this cancer. Similarly, in the same year, there were an estimated 480,000 cases of esophageal cancer and 400,000 deaths from this cancer. Esophageal cancer is the 8^th^ most common cancer and the 6^th^ most common cause of cancer death worldwide (8). The incidence of esophageal cancer varies greatly based on geographic area. The rate of this cancer is very high in the North of Iran. The present study was designed to assess the correlation between opium inhalation and laryngoesophageal cancers. 

## Materials and Methods

Fifty eight patients with laryngeal cancer, ninety five patients with esophageal cancer who had proven squamous cell carcinoma (SCC) through biopsy and twenty eight healthy individuals with no evidence of head and neck or esophageal malignancies who were matched for age were selected from Otolaryngology and Radiation Oncology Department at Mashhad University of Medical Sciences Between September 2008 to August 2010. Demographic information including age, sex, education, economic status and duration and amount of cigarette smoking and opium consumption by snuffing were recorded through a comprehensive interview. Opium use was defined as consuming the product at least once a day for a minimum of one year. 

*Statistical Analysis*

The Statistical Package for Social Sciences (SPSS version 13) was used for data analysis. Distribution of continuous variables was analyzed using the Kolmogorove Smirnov test for normality. Laryngeal and esophageal cancer were the outcomes of interest and opium dependency was the primary exposure. The confounding effects of sex, age and smoking were also assessed. Descriptive statistics (frequency, mean, and standard deviation) were determined for all variables. Baseline demographics and clinical characteristics were compared among groups using a student t-test, a Mann-Whitney test, a chi-square test, and/or a Fisher’s exact test as appropriate. For non- normal distribution of dependent variables, log linear analysis was done to adjust for confounders. The 95% confidence interval (CI) of the odds ratio (OR) was reported. A level of P<0.05 was reported as statistically significant. 

## Results

One hundred and eighty one cases and controls were entered into the study. The crude odds ratio for laryngeal cancer was 5.58 (95% CI 2.05-15.15, P=0.000) for cigarette smoking and 9.09 (95% CI 3.21-25.64, P=0.000) for opium dependency/consumption. The odds ratio for esophageal cancer was 0.44 (95%CI 0.18-1.09, P=0.07) for cigarette smoking and 1.44 (95%CI 0.57-3.62, P=0.43) for opium dependency. The odds ratio for opium dependency in laryngeal cancer after adjusting for smoking was still high with an OR of 6.06 (95% CI 1.10-33.23, P=0.05). Laryngeal cancer was detected at a significantly lower age in opium dependent patients (54.54±10.93 vs 62.92±10.10 years, P=0.02). This effect was not observed for smoking in laryngeal cancer patients and was also not detected in esophageal cancer patients. 

## Discussion

A number of studies have shown the effect of opium on different cancers. There is some evidence that opium has an effect on bladder, stomach, lung, esophageal and laryngeal cancers (3,4-7).The correlation between opium and laryngo-esophageal cancers is not well established. A variety of risk factors have been proposed in these two cancers, most notably cigarette smoking and alcohol use. Identifying other causative agents is important for preventive interventions to reduce the burden of these cancers. A recent study by Hakami et al. demonstrated that high cooking temperature and fried foods were associated with esophageal squamous cell carcinoma in high-risk areas of Iran (9). Previous studies from China and South America also suggest a correlation between hot beverage consumption and esophageal squamous cell cancer (10,11). The present study was designed to evaluate the impact of opium consumption on laryngeal and esophageal cancer. We found a significant correlation between both opium use and cigarette smoking and laryngeal cancer in unadjusted models. Interestingly, the crude OR for laryngeal cancer in opium users was approximately twice that of cigarette smokers. The correlation between opium use and laryngeal cancer remained significant even after adjusting for smoking (OR=6.06, P=0.05). These findings suggest that opium use is an independent risk factor for laryngeal cancer. Furthermore, our data indicates that the correlation between laryngeal cancer and opium consumption is stronger than cigarette smoking. The relatively strong correlation between opium and laryngeal cancer in our study is suggestive of a causal relationship. Moreover, the fact that this correlation was stronger than cigarette smoking, suggests that the effects of opium on carcinogenesis of the larynx is potentially more potent. To the best of our knowledge, there has been only one other report regarding opium dependency and laryngeal cancer, by Mousavi et al. from Iran (7). Our findings are consistent with that research, which also showed a strong association between opium dependency and laryngeal cancer even after adjusting for smoking. Like our study, those investigators also found a stronger correlation between laryngeal cancer and opium use than cigarette smoking. 

The age of onset or manifestation of laryngeal cancer is usually in the fifth to seventh decades of life. In the present study, the mean age of cancer presentation was 54 years for opium-dependent patients and 62 years for the non opium-dependent patients. This 8-year difference in age of diagnosis was significant. It was also consistent with Mousavi’s study where they found that opium dependent patients develop laryngeal cancer at a younger age than non-opium dependent patients. It is believed that opium use may trigger the onset of other forms of cancer at an earlier age. A study that examined the effects of opium on bladder cancer reported that opium addicts develop bladder cancer at a younger age than non-addicts (12).

We did not find a significant correlation between esophageal cancer and opium consumption. Our data are not consistent with a 2008 study by Nasrollahzadeh, where they found that the use opium and other derivates of opium were associated with higher risk of esophageal squamous cell carcinoma (6). It is unlikely that these inconsistencies are a result of inherent genetic differences between their study population and ours, since both studies were conducted in provinces that were within geographic proximity of each other in Northeastern Iran. We believe this discrepancy is likely a result of a weaker association between opium and esophageal cancer than laryngeal cancer. In fact, the authors of this study reported an OR of 2.12 (1.21-3.74) for opium use and laryngeal cancer, which is lower than our reported OR for opium and laryngeal cancer. As a result, this weaker correlation may be more difficult to demonstrate, especially in the absence of very large sample sizes. 

The mechanism by which opium causes laryngeal cancer is not entirely understood. Opium smoke and opium dross are thought to contain a number of compounds including aromatic hydrocarbons and nitrosamines, which have been shown to have mutagenic activity (13-14). It is also postulated that some of the alkaloid components mixed in with opium, such as morphine and codeine, have inhibitory effects on smooth muscle peristalsis. This slowed peristalsis may lead to prolonged exposure of the upper aerodigestive tract to carcinogens such as hot beverages and contribute to carcinogenesis (15). Our study has some limitations. Recall bias and measure bias are possibilities given that we had multiple interviewers and patients were asked to recall their opium and cigarette smoking habits. Potential confounders may have also influenced our results. However, the most significant confounder in this study is cigarette smoking, and we found that the association between opium use and laryngeal cancer remained significant even after we adjusted for smoking. 

## Conclusion

Opium addiction by snuffing is an independent risk factor for laryngeal cancer and not for esophageal cancer. Cigarette smoking increases this risk. Opium dependency can also increase the likelihood of laryngeal cancer at a lower age.
